# Genomic Characterization of the Mouse Ribosomal DNA Locus

**DOI:** 10.1534/g3.113.009290

**Published:** 2013-12-17

**Authors:** Gabriel E. Zentner, Stephanie A. Balow, Peter C. Scacheri

**Affiliations:** * Department of Genetics and Genome Sciences, Case Western Reserve University, 2109 Adelbert Road, Cleveland, Ohio 44106; † Comprehensive Cancer Center, Case Western Reserve University, 11000 Euclid Avenue, Cleveland, Ohio 44106

**Keywords:** rDNA, rRNA, ChIP-seq, Oct4, polycomb

## Abstract

The transcription of rRNA is critical to all living cells and is tightly controlled at the level of chromatin structure. Although the widespread adoption of genomic technologies including chromatin immunoprecipitation with massively parallel short-read sequencing (ChIP-seq) has allowed for the interrogation of chromatin structure on a genome-wide scale, until recently rDNA has not been analyzed by this technique. We extended genomic analysis of rDNA to mouse (*Mus musculus*), in which rDNA is similar in structure but highly divergent in sequence compared with human rDNA. Comparison of rDNA histone marks between mouse embryonic stem cells (mESCs) and more differentiated mouse cell types revealed differences between pluripotent and differentiated states. We also observed substantial divergence in rDNA histone modification patterns between mESCs and human embryonic stem cells (hESCs). Surprisingly, we found that the pluripotency factor OCT4 was bound to rDNA in similar patterns in mESCs and hESCs. Extending this analysis, we found that an additional 17 pluripotency-associated factors were bound to rDNA in mESCs, suggesting novel modes of rDNA regulation in pluripotent cells. Taken together, our results provide a detailed view of rDNA chromatin structure in an important model system and enable high-resolution comparison of rDNA regulation between mouse and human.

rRNA is central to cellular life. Its transcription accounts for more than half of all RNA synthesis in a growing cell (Moss *et al.* 2007) and is rate-limiting for ribosome biogenesis, thereby controlling protein synthesis and downstream processes, including cell proliferation. Disruption of rRNA synthesis and subsequent ribosome biogenesis are linked to pleiotropic growth defects in model organisms from yeast to mouse (Kongsuwan *et al.* 1985; Oliver *et al.* 2004; Deutschbauer *et al.* 2005; Azuma *et al.* 2006; Uechi *et al.* 2006; Danilova *et al.* 2008; Iwanami *et al.* 2008; Chakraborty *et al.* 2009) as well as to congenital anomaly syndromes and cancer in humans (Ridanpää *et al.* 2001; Ruggero and Pandolfi 2003; Ebert *et al.* 2008; Trainor *et al.* 2008; Zentner *et al.* 2010; Dauwerse *et al.* 2011).

In a given mammalian cell, several hundred copies of a single rDNA repeat are present (Prokopowich *et al.* 2003). The mammalian rDNA repeat (∼43 kb in human, ∼45.3 kb in mouse) is divided into two major portions: the coding region and intergenic spacer (IGS). The coding region, ∼13–14 kb in length, contains the sequences of the 18S, 5.8S, and 28S rRNA species as well as several noncoding transcribed spacer sequences. The IGS contains a large number of simple repeats, LINEs, SINEs, and ALU elements, and also harbors an enhancer, spacer promoter, and the core promoter of the adjoining rDNA repeat (Gonzalez and Sylvester 1995; Grozdanov *et al.* 2003). Within a given cell, only a fraction of the rDNA repeats are transcriptionally active and display a euchromatic chromatin structure characterized by histone modifications associated with transcriptional activity (*i.e.*, H3K4me, H3K9ac) and low levels of CpG methylation. The remainder of the repeats adopt a heterochromatic structure containing histone modifications associated with transcriptional repression (*i.e.*, H3K9me, H4K20me) and hypermethylation of CpG dinucleotides (McStay and Grummt 2008). RNA polymerase I (Pol I) transcribes the coding region of each active rDNA repeat into a precursor rRNA (pre-rRNA) transcript containing the 18S, 5.8S, and 28S rRNA sequences as well as the intervening noncoding spacers (Russell and Zomerdijk 2005). The pre-rRNA is subjected to a series of cleavage and chemical modification steps to yield the mature 18S, 5.8S, and 28S rRNA molecules, which are then assembled into ribosomes (Henras *et al.* 2008).

The analysis of rDNA chromatin structure by genomic approaches has been problematic because rDNA is not included in current genome assemblies and sequencing reads corresponding to rDNA are generally discarded during analysis. To facilitate genomic analysis of rDNA, we previously constructed a build of the human genome containing a single copy of rDNA to which we aligned short sequence reads from ChIP-seq and other genomic technologies (Zentner *et al.* 2011a). Using this approach, we described several findings of interest, including previously unknown regions of histone modification within rDNA and the association of the insulator-binding protein CTCF with rDNA. With this approach, active and silent rDNA repeats are sampled together in each ChIP; therefore, signals at rDNA represent an aggregate of signals from all immunoprecipitated repeats. This method could, in principle, be applied to any species for which a genome sequence and sequenced rDNA repeat are available.

Although the structure of the mouse and human rDNA repeats are quite similar, their nucleotide sequences are highly divergent (Gonzalez and Sylvester 1995; Grozdanov *et al.* 2003), potentially suggesting different modes of regulation. To explore this possibility, we extended our previously described method of aligning high-throughput sequencing data to a genome build containing rDNA to the mouse. Using previously generated ChIP-seq data, we analyzed the distribution of histone modifications at rDNA in mouse embryonic stem cells (mESCs), mESC-derived neural precursor cells (mNPCs), and mouse embryonic fibroblasts (MEFs). We found that patterns of rDNA histone modifications in mESCs showed differences from those in mNPCs and MEFs, as well as human embryonic stem cells (hESCs). Strikingly, we found the pluripotency factor OCT4 associated with rDNA in mESCs and hESCs. Extending this analysis further, we observed rDNA association of an additional 14 pluripotency factors as well as three Polycomb proteins in mESCs, suggesting previously unsuspected mechanisms of rDNA regulation. Our results provide insight into chromatin-level regulation of rDNA in an important model organism and allow for comparison of rDNA regulation between human and mouse.

## Materials and Methods

### Datasets

The following datasets were obtained from the SRA: mESC H3K4me1, and H3K4me2 (SRP000230) (Meissner *et al.* 2008); H3K4me3, H3K9me3, H3K27me3, H3K36me3, H4K20me3, and input (SRP000415) (Mikkelsen *et al.* 2007); OCT4, SOX2, NANOG, SMAD1, STAT3, KLF4, c-MYC, n-MYC, and ZFX (SRP000217) (Chen *et al.* 2008); CHD7 and P300 (SRP0002695) (Schnetz *et al.* 2010); BRG1 (SRX003888) (Ho *et al.* 2009); EZH2, SUZ12, and RING1B (SRP000711) (Ku *et al.* 2008); nGFP (SRX207161) (Yamaji *et al.* 2013); CDX2 (SRX012415) (Nishiyama *et al.* 2009); SOX17 (SRX214076) (Aksoy *et al.* 2013); TBX3 (SRP001585) (Han *et al.* 2010); ZC3H11A (SRX188830) and RNA-seq (SRX019275) (Guttman *et al.* 2010); mNPC H3K4me1 and H3K4me2 (SRP000230) (Meissner *et al.* 2008); H3K4me3, H3K9me3, H3K27me3, H3K36me3, and input (SRP000415) (Mikkelsen *et al.* 2007); MEF H3K4me1 (SRX085451), H3K4me3, H3K9me3, H3K27me3, H3K36me3, and input (SRP000415) (Mikkelsen *et al.* 2007); and hESC OCT4 (SRP002512) and input (SRP003670) (Rada-Iglesias *et al.* 2011).

### Alignment and analysis of sequencing data

We used our previously described framework for the alignment of sequencing tags to a build of the MM8 genome assembly containing a single mouse rDNA repeat (GenBank accession no. BK000964) added to chromosome 12 (MM8_plus_rDNA) (Zentner *et al.* 2011a). Briefly, unique reads were aligned to the MM8_plus_rDNA genome assembly with Bowtie (Langmead *et al.* 2009), allowing two or fewer mismatches per read and discarding reads with more than one reportable alignment. Peaks were detected with F-seq (Boyle *et al.* 2008) using a fragment size of 200 bp and visualized in R after subtracting input signal at each base. We compared the intensity of ChIP-seq signals at rDNA to those along chromosome 12 via normalized tag density, which was calculated by dividing the mean intensity of all peaks called within rDNA by the mean intensity of all peaks called outside of rDNA on chromosome 12. For correlation analyses, the rDNA locus was divided into 100-bp bins and the median signal in each bin was determined. Least-squares regression was performed using the lm function of R and the associated F-test *P* value was reported. Heatmaps were generated using the gplots R package. Two hESC OCT4 ChIP-seq technical replicates were concatenated, aligned to HG18_plus_rDNA, and analyzed as described (Zentner *et al.* 2011a).

The mappability of mouse rDNA was assessed using bias elimination algorithm for deep sequencing (BEADS) (Cheung *et al.* 2011). Mouse rDNA was divided into 36-bp fragments at 1-bp intervals and fragments were aligned to MM8_plus_rDNA as described. Mapped reads were extended to 200 bp in the 5′ and 3′ directions to simulate the estimated 200-bp fragment size. Extended fragments were concatenated and mappability was defined as the number of fragments overlapping each base. Because reads may be positive-stranded or negative-stranded, the maximum number of 200-bp fragments that can overlap a given genomic position is 400 (100% mappability). The mappability value for each base of mouse rDNA is presented in Supporting Information, Table S1. Base positions with a mappability of <25% were considered to be poorly mappable because they were discarded from analysis in the original BEADS study (Cheung *et al.* 2011).

### ChIP

The mESCs and mNPCs were cultured as previously described (Schnetz *et al.* 2009). ChIP was performed as described (Schmidt *et al.* 2009) from 5 × 10^6^ to 1 × 10^7^ crosslinked cells. PCR reactions were performed in triplicate on the ABI7300 real-time PCR system using Sybr Green chemistry (ABI). The following antibodies were used for ChIP: rabbit anti-H3K4me1 (8 µg/ChIP; Abcam #8895) and goat anti-OCT4 (10 µg/ChIP; Santa Cruz #8628). Primers used for ChIP-PCR are detailed in Table S2.

### qRT-PCR

RNA was extracted from mESCs and mNPCs using TRIzol (Invitrogen), and cDNA was prepared using the High-Capacity cDNA Archive Kit (ABI). Triplicate PCR reactions using Sybr Green (ABI) on an ABI 7300 real-time thermal cycler. *GAPDH* was used for endogenous control qRT-PCR reactions. Primers used for qRT-PCR are detailed in Table S3.

## Results

### Analysis of sequencing data

To analyze mouse rDNA by ChIP-seq, we constructed a build of the mouse genome containing a single copy of the mouse rDNA repeat. The mouse rDNA repeat was added to the proximal end of chromosome 12, on which rDNA is located endogenously, to allow comparison of rDNA signals to those on nucleoplasmic chromatin. This genome build was designated “MM8_plus_rDNA.” We discarded duplicate reads and reads aligning to multiple locations in the genome to reduce false-positives. After alignment, experimental data were normalized to input genomic DNA to correct for systematic biases.

**Figure 1:**
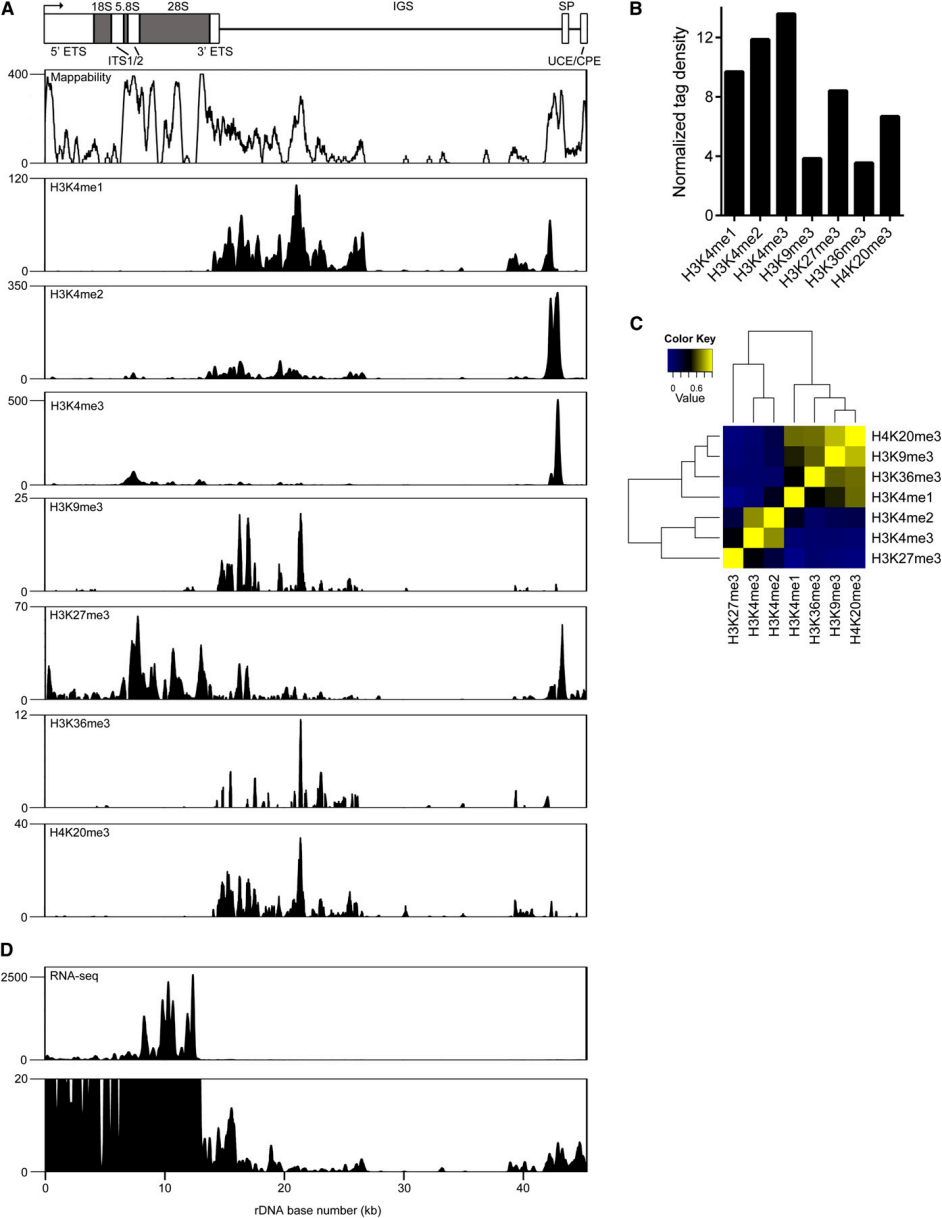
Distribution of histone modifications on rDNA in mESCs. (A) Schematic representation of the mouse rDNA repeat based on GenBank accession no. BK000964. The rDNA TSS is depicted by an arrow. ETS, external transcribed spacer; ITS, internal transcribed spacer; IGS, intergenic spacer; SP, spacer promoter; UCE, upstream control element; CPE, core promoter element. Below the schematic is a mappability track for mouse rDNA as determined by BEADS (Cheung *et al.* 2011), with the mappability score for each base plotted on the Y-axis (maximum mappability = 400). Presented below the mappability track are ChIP-seq plots showing the distributions of H3K4me1, H3K4me2, H3K4me3, H3K9me3, H3K27me3, H3K36me3, and H4K20me3 at rDNA in mESCs. Signal intensity as determined by F-seq is plotted on the Y-axis. (B) Normalized tag density scores for histone modifications at rDNA in mESCs. (C) Heatmap of correlation scores for pairwise comparisons between the median signals for each histone modification at rDNA in mESCs. (D) RNA-seq profiles across rDNA in mESCs. Signal intensity as determined by F-seq is plotted on the Y-axis. Shown below is an RNA-seq plot with a reduced Y-axis scale to display low-intensity RNA-seq signals.

We also analyzed the mappability of mouse rDNA because there are many rDNA pseudogenes throughout the genome that might complicate analysis (Gonzalez and Sylvester 1997). Mappability is a measure of the uniqueness of a given genomic sequence based on the number of sequenced fragments uniquely alignable to that sequence. We analyzed the mappability of mouse rDNA using BEADS (Cheung *et al.* 2011), a bias-correction software suite for sequencing data. We divided the rDNA into 36-mers at 1-bp intervals and mapped these fragments to MM8_plus_rDNA as described. We then extended the mapped reads to 200 bp to match our estimated fragment size and determined the number of fragments overlapping each base. Regions of robust mappability were detected within the coding region, at the IGS from ∼14 kb to 26 kb, and at the spacer promoter. Poorly mappable regions (those defined as having a mappability <25%) (Cheung *et al.* 2011) were found from ∼2 kb to 5 kb of the rDNA coding region and at various points throughout the 18S coding region, as well as the IGS. A region of the IGS from ∼27 kb to 42 kb showed particularly poor mappability (Figure 1A), likely a result of the high repeat content of this region (Grozdanov *et al.* 2003). Overall, 13,371 out of 45,309 (29.5%) bases in the mouse rDNA repeat displayed a mappability >25%. The coding region displayed a substantially higher overall mappability (45.3%), whereas the IGS showed generally lower mappability (22.8%).

### Distribution of histone modifications at rDNA in mESCs

We first examined the distribution of seven histone modifications on rDNA (depicted schematically in Figure 1A; this schematic is included above all rDNA plots for reference) in mESCs: H3K4me1, generally associated with enhancers (Barski *et al.* 2007; Heintzman *et al.* 2007, 2009; Ernst *et al.* 2011; Rada-Iglesias *et al.* 2011; Zentner *et al.* 2011b); H3K4me2, found at promoters and enhancers (Barski *et al.* 2007; Lupien *et al.* 2008; Ernst *et al.* 2011); H3K4me3, enriched at transcription start sites (TSSs) (Barski *et al.* 2007; Heintzman *et al.* 2007; Ernst *et al.* 2011); H3K9me3 and H4K20me3, associated with heterochromatin (Mikkelsen *et al.* 2007); H3K27me3, the signature of Polycomb repression (Cao *et al.* 2002; Müller *et al.* 2002); and H3K36me3, primarily associated with the bodies of transcribed genes (Vakoc *et al.* 2006). We detected three major areas of histone modification enrichment: within the coding region, which spans 0–13.4 kb of the repeat; a broad region from ∼14 kb to 26 kb; and proximal to the spacer promoter, located ∼2 kb upstream of the rDNA TSS. Notably, these are regions of high mappability (Figure 1A).

Within the coding region, we detected modest enrichment of H3K4me2 and H3K4me3 at ∼9 kb into the repeat. H3K27me3 was also broadly distributed over this region, potentially indicating Polycomb repression of a subset of rDNA repeats (Figure 1A). Within the broad region from ∼14 kb to 26 kb, several modifications were detected. H3K4me1 was robustly enriched throughout this region, with particularly strong enrichment at ∼21 kb. H3K4me2 followed a similar distribution in this region, but with less robust signal intensity, whereas H3K4me3 was virtually absent. Varying degrees of enrichment of H3K9me3, H3K36me3, and H4K20me3 were also detected in this region, with maximal signal generally seen at ∼21 kb. H3K27me3 showed some regions of moderate enrichment within this region (Figure 1A). At the spacer promoter, all methylated forms of H3K4 as well as H3K9me3 were detected, reflecting the mixture of active and silent rDNA repeats sampled in these ChIP experiments (Figure 1A).

Given the high copy number of rDNA, it might be expected that signals at rDNA would appear inflated compared with those on nucleoplasmic chromatin when reads are aligned to a genome build containing a single rDNA repeat. However, many factors may influence the signal at rDNA, including the numbers of active and silent repeats, ChIP efficiency, and signal scaling at high copy sequences by peak calling algorithms. Nevertheless, we would expect rDNA signals to be at least as intense as those on nucleoplasmic chromatin. We compared the intensity of histone modification signals at rDNA to those along chromosome 12 by generating a normalized tag density score for each mark (see *Materials and Methods*). Histone modification signals were ∼3.5–13.6 times as intense as those seen along chromosome 12 (Figure 1B).

We next determined the median signal for each mark across rDNA in 100-bp windows and performed pairwise correlation analysis for the entire locus. Two primary clusters were identified, one containing H3K27me3, H3K4me2, and H3K4me3 and the other containing H4K20me3, H3K9me3, H3K36me3, and H3K4me1 (Figure 1C). This is in contrast to human rDNA, in which modifications associated with transcriptional activation and repression separated into two distinct groups (Zentner *et al.* 2011a). However, within each cluster, highly correlated pairs of modifications associated with transcriptional activity or silencing were detected (H3K4me2 and H3K4me3; H3K9me3 and H4K20me3) (Figure 1C).

The coding region of active rDNA repeats is highly transcribed, containing a very high density of Pol I molecules (∼1 molecule/100 bp) (Grummt 1999), and rRNA comprises much more than half of all RNA synthesized by a given cell (Moss *et al.* 2007). We therefore assessed transcription from rDNA using RNA-seq data from mESCs (Guttman *et al.* 2010). Despite using data generated from PolyA-selected RNA, we were able to detect RNA-seq signal originating from rDNA. Very high levels of RNA-seq signal were detected over the coding region of rDNA, consistent with an extremely high volume of Pol I transcription (Figure 1D). In addition to transcription of the rDNA coding region, it has been demonstrated that the rDNA spacer promoter, located ∼2 kb upstream of the core promoter region, directs transcription of short noncoding RNA molecules. These ncRNAs are homologous the rDNA core promoter and have been designated promoter-associated RNA (pRNA). pRNA appears to function in the epigenetic regulation of the rDNA locus (Bierhoff *et al.* 2011). We therefore expanded the scale of our RNA-seq data to determine the presence of pRNA and any other low-abundance RNA species originating from rDNA. We observed RNA-seq signal at ∼43 kb and ∼45 kb into the repeat, indicating the presence of pRNA corresponding to the spacer and core promoter regions (Figure 1D). Several other regions of RNA-seq enrichment outside of the coding region were also detected, although their significance is unclear at this time.

### rDNA histone modification patterns in differentiated mouse cell types

**Figure 2:**
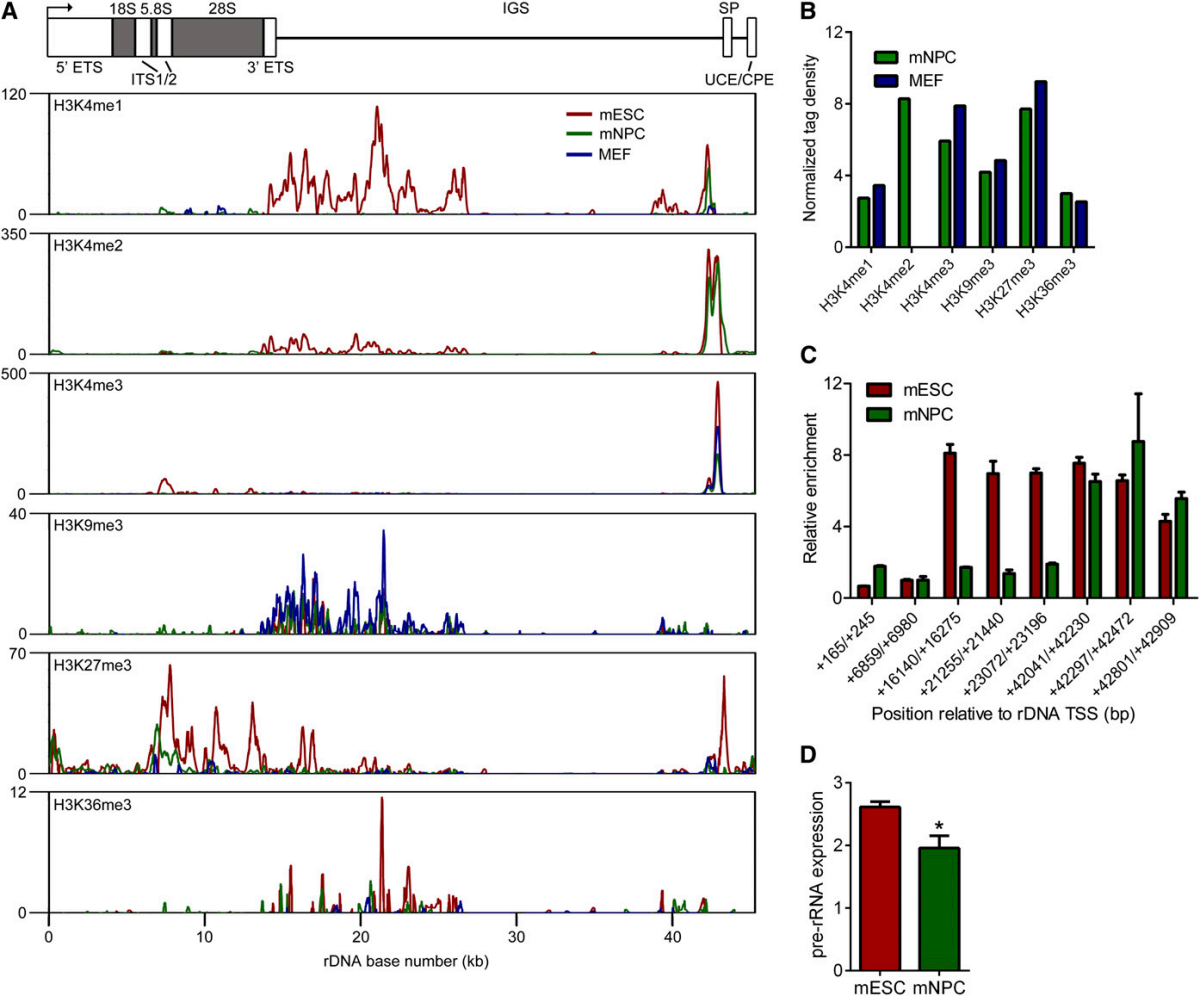
Comparison of rDNA histone modifications between mESCs and mNPCs. (A) ChIP-seq plots comparing the distributions of H3K4me1, H3K4me2, H3K4me3, H3K9me3, H3K27me3, and H3K36me3 at rDNA in mESCs, mNPCs, and MEFs. Signal intensity as determined by F-seq is plotted on the Y-axis. (B) Normalized tag density scores for histone modifications at rDNA in mNPCs and MEFs. (C) ChIP-PCR evaluation of H3K4me1 enrichment at rDNA in mESCs and mNPCs. Enrichment values were normalized to the +6859/+6980 negative control amplicon. Error bars represent mean ± SD for triplicates. (D) Pre-rRNA levels in mESCs and mNPCs as measured by qRT-PCR. Mean ± SEM is shown (n = 2). *P = 0.045 by one-tailed *t* test.

We next tried to determine if the distribution of histone modifications at rDNA differed between undifferentiated and differentiated cell types. To this end, we compared rDNA histone modification patterns between mESCs, mESC-derived mNPCs, and MEFs visually and by pairwise linear regression for the entire locus. mNPCs and MEFs lost enrichment of H3K4me1 in the ∼14-kb to 26-kb region of enrichment, potentially suggesting that the activity of any functional element within this region is restricted in the mESC state (Figure 2A). This drastic reduction in H3K4me1 in mNPCs and MEFs likely contributed to the lack of correlation between its locus-wide distributions (mESC *vs.* mNPC: *R^2^* = 0.04, *P* = 2.08 × 10^−5^; mESC *vs.* MEF: *R^2^* = 0.005, *P* = 0.271). Visually, H3K4me2 also seemed to be lost at this region, but the major site of enrichment, at the spacer promoter, was unaffected, and the locus-wide correlation remained robust and highly significant (mESC *vs.* mNPC: *R^2^* = 0.87, *P* <2.2 × 10^−16^). H3K4me3 also appeared to decrease at the spacer promoter region, but its locus-wide distribution was not affected (mESC *vs.* mNPC: *R^2^* = 0.93, *P* < 2.2 × 10^−16^; mESC *vs.* MEF: *R^2^* = 0.94, *P* < 2.2 × 10^−16^). The locus-wide distributions of H3K9me3 and H3K27me3 were largely correlated between cell types, with the exception of MEF H3K27me3 (mESC *vs.* mNPC H3K9me3: *R^2^* = 0.61, *P* < 2.2 × 10^−16^; mESC *vs.* mNPC H3K27me3: *R^2^* = 0.58; mESC *vs.* MEF H3K9me3: *R^2^* = 0.66, *P* < 2.2 × 10^−16^; mESC *vs.* MEF H3K27me3: *R^2^* = 0.1, *P* = 5.98 × 10^−12^). The distributions of H3K36me3 were not correlated between mESCs, mNPCs, and mESCs (mESC *vs.* mNPC: *R^2^* = −0.002, *P* = 0.58; mESC *vs.* MEF: *R^2^* = −0.002, *P* = 0.69). Normalized tag densities for mNPC and MEF histone marks ranged from ∼2.5 to 9.2 (Figure 2B). Using ChIP-PCR we confirmed that H3K4me1 was reduced within the IGS but maintained at the spacer promoter in mNPCs (Figure 2C).

Because H3K4me1 decreases at rDNA during the transition from the mESC to mNPC state, we wondered if rRNA expression might also decrease. To this end, we measured the expression of the pre-rRNA transcript in mESCs and mNPCs by qRT-PCR. We observed a significant decrease in pre-rRNA expression (*P* = 0.045 by one-tailed *t* test) in mNPCs *vs.* mESCs (Figure 2D). We speculate that the reduction in H3K4me1 within the spacer promoter in mNPCs may be linked to reduced rRNA expression, although we cannot rule out the influence of other histone modifications or cell type–specific transcription factors not assayed here.

### Differences in histone modification patterns at mESC and hESC rDNA

**Figure 3:**
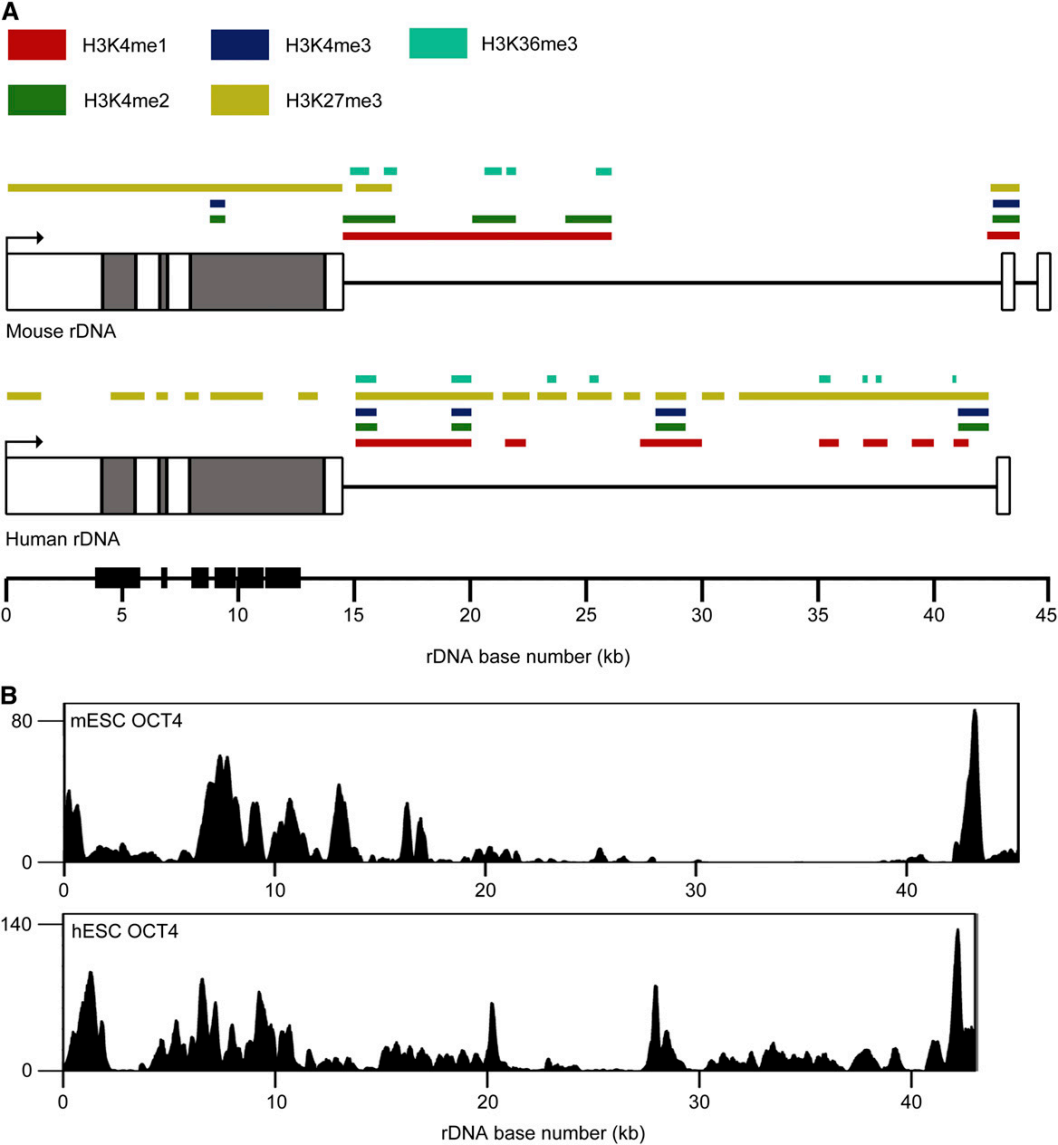
Comparison of rDNA histone modifications between mESCs and hESCs. (A) Schematic representations of the mouse and human rDNA repeats showing sites of histone modification enrichment in mESCs and hESCs as colored boxes. Repeats are depicted approximately to scale (mouse, ∼45 kb; human, ∼43 kb). Regions of high sequence identity as determined by two-sequence BLAST are depicted as black boxes on the scale bar. (B) ChIP-seq profiles of OCT4 at rDNA in mESCs and hESCs. Signal intensity as determined by F-seq is plotted on the Y-axis.

Despite the highly similar structures of mouse and human rDNA, their sequences are highly divergent. We previously analyzed the distributions of several histone modifications on rDNA in hESCs (Zentner *et al.* 2011a). Using these data, we compared patterns of rDNA histone modification in mESCs to those in hESCs. A schematic of the distributions of five histone modifications analyzed in both cells types is presented in Figure 3A. Within the coding region of both mouse and human rDNA, substantial H3K27me3 enrichment was seen; however, the enrichment of H3K4me2 and H3K4me3 seen at ∼9 kb in the mouse rDNA repeat was not observed in human rDNA and, overall, H3K27me3 had a broader distribution on human rDNA, encompassing nearly the entire repeat. H3K4me1 enrichment was seen from ∼14 kb to 20 kb in the mouse and human repeats, although it extended to ∼26 kb in mouse. Whereas discrete peaks of H3K4me2 and H3K4me3 were seen at ∼15 kb and ∼20 kb in the human repeat, these peaks were not evident in mouse rDNA, although the ∼21-kb peak of H3K4me1 in mouse rDNA may correspond to this latter peak. No H3K4me3 enrichment was seen in this region of mouse rDNA. The peak of activation-associated modifications seen at ∼27–28 kb in human rDNA was not found in mouse rDNA. Several regions of H3K4me1 and H3K36me3 enrichment in the human IGS were not observed in the mouse IGS. Methylation of H3K4 and H3K9me3 were detected at the spacer promoter region in human and mouse rDNA. Overall, it appears that the distributions of histone marks at rDNA are quite different between mESCs and hESCs.

Given the marked differences in rDNA histone modifications between mESCs and hESCs, we wondered if other factors known to differentially occupy the genome in mESCs and hESCs might also bind rDNA in distinct patterns. We chose to analyze ChIP-seq data for the key pluripotency factor OCT4, which has distinct genomic distributions in mESCs and hESCs (Loh *et al.* 2006) and for which there is some evidence of nucleolar localization (Table 1). Strikingly, comparison of OCT4 ChIP-seq profiles in mESCs and hESCs revealed relatively similar distributions of OCT4, with high enrichment across the coding region and proximal to the spacer promoter (Figure 3B). A notable exception is a peak of OCT4 enrichment at ∼27–28 kb into the human rDNA repeat not seen in mouse rDNA. This region is also enriched for active histone modifications in hESCs (Figure 3A) (Zentner *et al.* 2011a). The overall similarity in OCT4 rDNA binding between mESCs and hESCs is surprising given that OCT4 tends to occupy distinct sites in mESCs and hESCs (Loh *et al.* 2006) and suggests that pluripotency factors may regulate rRNA expression.

**Table 1:** Evidence for nucleolar localization of pluripotency factors in mESCs

**Protein**	**Evidence**	**Cell Types**
OCT4	Nucleolar localization (Parfenov *et al.* 2003; Zuccotti *et al.* 2008); interacts with CHD4, a known regulator of rRNA synthesis (Shimono *et al.* 2005; van den Berg *et al.* 2010); interacts with nucleolar protein nucleophosmin (Johansson and Simonsson 2010)	Mouse oocytes, mESCs
SOX2	Predicted to be nucleolar based on sequence motifs (Bauer *et al.* 2011); interacts with nucleolar protein nucleophosmin (Johansson and Simonsson 2010)	mESCs
NANOG	Nucleolar localization (He *et al.* 2006); interacts with nucleolar protein nucleophosmin (Johansson and Simonsson 2010)	ICM of goat embryos, mESCs
KLF4	Not available	Not available
STAT3	Not available	Not available
SMAD1	Interacts with RUNX2, a known regulator of rRNA synthesis (Zhang *et al.* 2000; Ali *et al.* 2008)	NIH3T3, P19 cell lines
c-MYC	Nucleolar localization and regulation of rRNA transcription (Poortinga *et al.* 2004; Arabi *et al.* 2005; Grandori *et al.* 2005)	WI38, COS-7, HeLa, U2OS, Rat1, CHO, NIH-3T3, HL-60, MPRO cell lines, human primary foreskin fibroblasts
n-MYC	Predicted to be nucleolar based on sequence motifs (Bauer *et al.* 2011)	Not available
ZFX	Not available	Not available
E2F1	Nucleolar localization on ARF expression (Datta *et al.* 2002)	U2OS cell line
ESRRB	Interacts with CHD4, a known regulator of rRNA synthesis (Shimono *et al.* 2005; van den Berg *et al.* 2010)	mESCs
TCFCP2L1	Interacts with CHD4, a known regulator of rRNA synthesis (Shimono *et al.* 2005; van den Berg *et al.* 2010)	mESCs
P300	Closely related protein CBP acetylates Pol I transcription factor UBF to stimulate rRNA transcription (Pelletier *et al.* 2000); interacts with CHD7, a known regulator of rRNA synthesis (Schnetz *et al.* 2010; Zentner *et al.* 2010)	NIH3T3 cell line
BRG1	Interacts with CHD4 and CHD7, known regulators of rRNA synthesis (Shimono *et al.* 2003; Bajpai *et al.* 2010; Zentner *et al.* 2010)	HEK293 cell line, human neural crest–like cells

### Pluripotency factors associated with rDNA in mESCs

**Figure 4:**
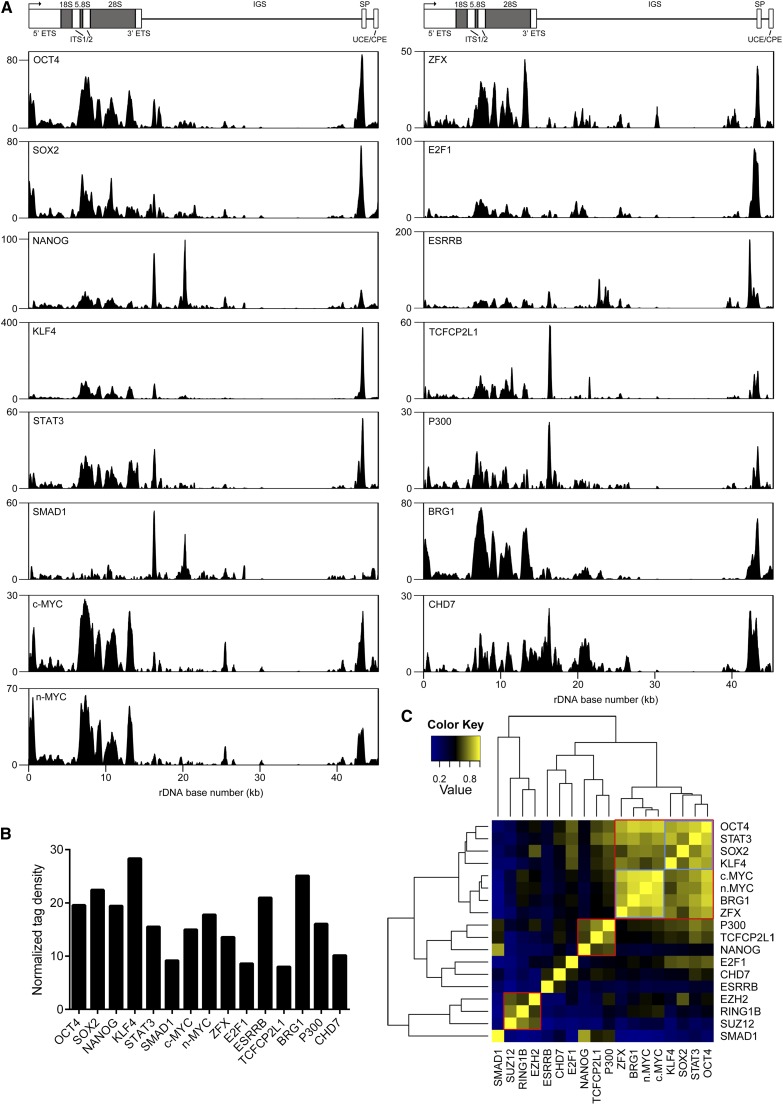
mESC pluripotency factors are associated with rDNA. (A) ChIP-seq profiles of OCT4, SOX2, NANOG, KLF4, STAT3, SMAD1, c-MYC, n-MYC, ZFX, E2F1, ESRRB, TCFCP2L1, P300, BRG1, and CHD7 at rDNA in mESCs. Signal intensity as determined by F-seq is plotted on the Y-axis. (B) Normalized tag density scores for pluripotency factors at rDNA in mESCs. (C) Heatmap of pairwise correlation scores between pluripotency factors and Polycomb proteins at rDNA.

Having determined that OCT4 was associated with rDNA in mESCs and hESCs, we sought to determine if additional pluripotency-associated factors might be bound to rDNA. We obtained ChIP-seq datasets for a number of well-established components of the mESC pluripotency network, SOX2, NANOG, KLF4, STAT3, SMAD1, C-MYC, N-MYC, ZFX, E2F1, ESRRB, TCFCP2L1 (Chen *et al.* 2008), P300 (Schnetz *et al.* 2010), and BRG1 (Ho *et al.* 2009), and aligned them to MM8_plus_rDNA. We also reanalyzed CHD7 mESC ChIP-seq data (Schnetz *et al.* 2010), which we previously aligned to mouse rDNA out of the context of the reference genome (Zentner *et al.* 2010), after alignment to MM8_plus_rDNA. Strikingly, we found that all analyzed factors were associated with rDNA (Figure 4A). We generated normalized tag density scores for each factor and found that the enrichment of each protein at rDNA was more than eight-fold higher than its enrichment along chromosome 12 (Figure 4B). ChIP-PCR confirmed the association of OCT4 with rDNA (Figure S1). Notably, we also found additional experimental evidence of nucleolar localization for nearly all of the tested factors (Table 1).

To ensure that the observed binding of pluripotency factors to rDNA was not simply attributable to the highly accessible chromatin architecture and high number of active rDNA repeats, we first analyzed ChIP-seq data for nuclear GFP (nGFP) (Yamaji *et al.* 2013), because GFP has been shown to give artifactual ChIP signals at highly expressed loci (Teytelman *et al.* 2013). We noted some GFP ChIP signal at the 5′ end of the repeat (Figure S2), but closer inspection revealed that this region showed an irregular, jagged distribution of tags rather than the smooth gradation of tags generally seen with legitimate peak; therefore, we speculate that this is an artifact. We also analyzed ChIP-seq datasets for the CDX2, TBX3, SOX17, and ZC3H11A transcription factors (Nishiyama *et al.* 2009; Han *et al.* 2010; Aksoy *et al.* 2013). Similar to GFP, we noted irregular enrichment at the 5′ end of the repeat as well as a highly discrete region of enrichment at ∼15.7 kb in each of the negative control TF datasets (Figure S2). Given that this peak is precisely overlapping between each of the negative control datasets and does not overlap with binding sites for pluripotency factors, we speculate that it is an artifact. From these analyses, we are confident that the association of pluripotency factors with rDNA is legitimate.

**Figure 5:**
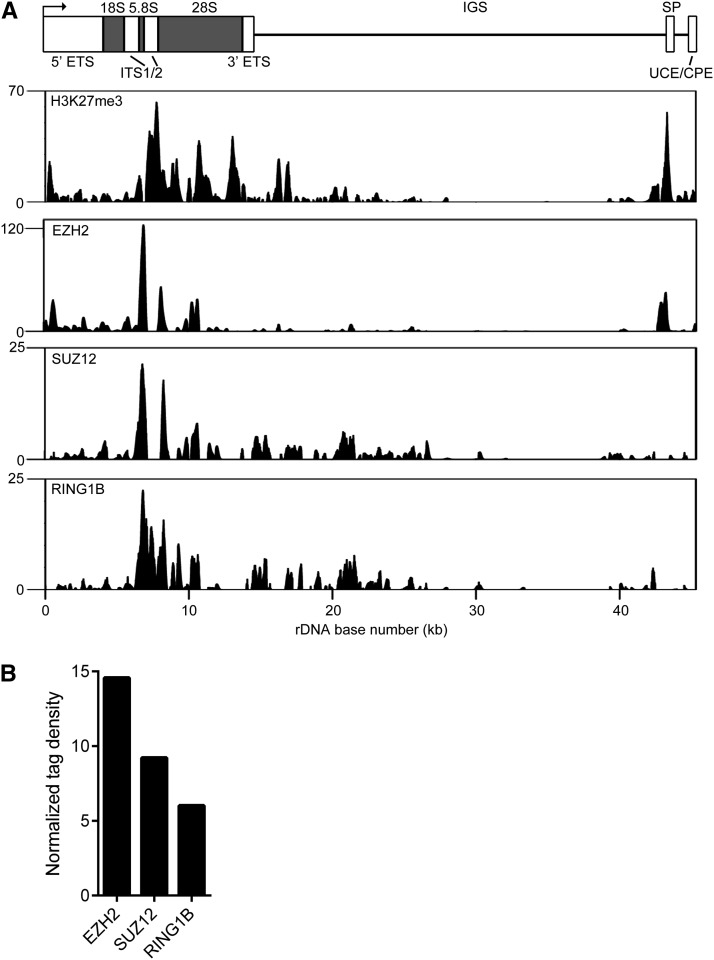
Polycomb proteins bind rDNA. (A) ChIP-seq profiles of H3K27me3, EZH2, SUZ12, and RING1B enrichment at rDNA in mESCs. Signal intensity as determined by F-seq is plotted on the Y-axis. (B) Normalized tag density scores for Polycomb proteins at rDNA in mESCs.

In the nucleoplasm, several pluripotency factors often bind to the same genomic regions, designated multiple transcription factor loci (MTLs) (Chen *et al.* 2008). Two major classes of MTLs have been described: an “OCT4-centric” module containing OCT4, SOX2, NANOG, STAT3, SMAD1, and other factors, and a “Myc-centric” module containing c-MYC, n-MYC, E2F1, and ZFX, among other proteins (Chen *et al.* 2008; Schnetz *et al.* 2010; Ng and Surani 2011). We were curious about whether such MTLs might be detected at rDNA. Therefore, we performed correlation analysis for the aforementioned analyzed factors plus Polycomb proteins (Figure 5). This analysis revealed several groups of factors (Figure 4C). One major cluster (top right, outlined in red in Figure 4) comprised two smaller groups, one containing OCT4, STAT3, SOX2, and KLF4 (outlined in blue in Figure 4) and the other containing c-MYC, n-MYC, BRG1, and ZFX (outlined in blue in Figure 4). Notably, NANOG, a component of the core pluripotency machinery, did not cluster with factors such as OCT4 and SOX2; rather, NANOG clustered with P300 and TCFCP2L1 (center, outlined in red in Figure 4). As expected, the Polycomb group proteins EZH2, SUZ12, and RING1B strongly colocalized throughout the rDNA repeat (bottom left, outlined in red in Figure 4). Although SMAD1 did not belong to any distinct cluster, it showed some degree of correlation with NANOG, TCFCP2L1, and P300, with which it shared a somewhat similar signal distributions (Figure 4A). In general, the groups of factors we detected at rDNA were quite similar to those seen on nucleoplasmic chromatin (Chen *et al.* 2008; Schnetz *et al.* 2010; Ng and Surani 2011).

### Polycomb proteins bind rDNA

In this study, we have shown that mouse rDNA is marked by H3K27me3, the chromatin signature of Polycomb-mediated transcriptional repression. We also previously showed that this mark is present throughout the human rDNA repeat in hESCs (Figure 3A) (Zentner *et al.* 2011a). We therefore speculated that Polycomb proteins might be associated with rDNA. To assess this possibility, we made use of previously published mESC ChIP-seq data for the Polycomb group proteins EZH2, SUZ12, and RING1B (Ku *et al.* 2008). We detected binding of these three factors to rDNA in a pattern highly similar to that of H3K27me3 (Figure 5A) (*R^2^* = 0.39−0.58, *P* < 2.2 × 10^−16^ for all), further suggesting that Polycomb silencing is involved in the maintenance of transcriptional silencing at rDNA and indicating that the results of the H3K27me3 ChIP-seq analysis are legitimate. The normalized tag densities for Polycomb proteins were ∼6.0–14.6 (Figure 5B).

## Discussion

The rDNA is a critical genomic region tightly controlled at the level of chromatin structure. Here, we aligned sequencing data from ChIP-seq and other genomic technologies to a build of the mouse genome containing a single rDNA repeat. We provide several lines of evidence that the signals we observed at rDNA are legitimate. First, the ChIP-seq signals we observed fall into regions of high mappability. Second, consistent with the high copy number of rDNA, the ChIP-seq signals at rDNA were all higher than those observed on nucleoplasmic chromatin. Third, the ChIP-seq signals were validated by standard ChIP. Fourth, we failed to detect rDNA association of nGFP or several transcription factors, arguing for specific association of pluripotency factors with rDNA in mESCs. Finally, H3K27me3 enrichment at rDNA coincides with enrichment of the Polycomb proteins EZH2, SUZ12, and RING1B, which are known to contribute to deposition of the H3K27me3 mark and show colocalization with H3K27me3 on nucleoplasmic chromatin.

Our results highlight similarities and substantial differences in the chromatin-level regulation of rDNA between mouse and human. Enrichment of activation and repression-associated marks ∼2 kb upstream of the core promoter region is common to both mESCs and hESCs, suggesting that chromatin-level regulation of pRNA production is conserved. The differences in histone modification distributions between mESC and hESC rDNA may reflect species-specific differences or may indicate differences in developmental stage, as previously proposed (Brons *et al.* 2007; Tesar *et al.* 2007). It is also possible that the differences in histone modification patterns seen between mouse and human rDNA could be attributable to the high degree of divergence in the genetic architecture of the repeat between the two species (Gonzalez and Sylvester 1995; Grozdanov *et al.* 2003). Regardless, we speculate that the regions of histone modification in both the mouse and human IGSs are reflective of novel functional elements.

The association of pluripotency factors, including OCT4 and SOX2, with rDNA is particularly interesting. Factors such as MYOD and RUNX2, important for myogenic and osteogenic differentiation, respectively, also associate with rDNA and downregulate rRNA expression during differentiation. Therefore, it has been suggested that a general property of lineage-specific transcription factors is regulation of rRNA transcription as a means to coordinate cell growth and phenotype (Ali *et al.* 2008). Extending this hypothesis to mESCs, it might be speculated that factors that maintain mESC identity serve in the place of lineage-specific transcription factors in regulating rRNA expression. We found that more than a dozen pluripotency-associated factors were bound to rDNA. With some exceptions (*i.e.*, the lack of correlation between NANOG and OCT4/SOX2), the clusters of factors we observed at rDNA (“rDNA MTLs”) were remarkably similar to those in the nucleoplasm; for instance, OCT4 and SOX2 were strongly correlated, as were C-MYC, N-MYC, and ZFX. This suggests that pluripotency factors function to regulate transcription in similar combinations in both the nucleoplasm and nucleolus.

The association of Polycomb proteins with rDNA in mESCs is also intriguing, suggesting that Polycomb proteins silence rDNA, similar to their function in the nucleoplasm. Polycomb proteins are repressors of nucleoplasmic gene transcription (Bannister and Kouzarides 2011), and perhaps they also participate in the repression of Pol I transcription. An additional nonmutually exclusive possibility is that Polycomb proteins may also function in the suppression of cryptic transcription of rDNA and rDNA recombination, both of which have deleterious cellular consequences in yeast and mammalian cells (Kobayashi and Ganley 2005; Gagnon-Kugler *et al.* 2009). Again, functional studies are needed to assess these possibilities.

It has been appreciated for some time that the cell uses common epigenetic mechanisms to regulate both Pol I and Pol II transcription (*i.e.*, DNA methylation, histone modifications, chromatin remodeling) (McStay and Grummt 2008). However, there is also accumulating evidence for a large class of transcription factors and chromatin remodelers that dually function in nucleoplasmic and nucleolar transcription regulation (*i.e.*, MYOD, C-MYC, CHD4/7) (Arabi *et al.* 2005; Grandori *et al.* 2005; Shimono *et al.* 2005; Ali *et al.* 2008; Zentner *et al.* 2010). This emerging theme is underscored by our finding that more than a dozen pluripotency-associated transcription factors and chromatin remodelers associate with rDNA in mESCs. Therefore, it may be speculated, despite the distinctive machineries that transcribe rRNA and nucleoplasmic genes, that the cell uses generally similar mechanisms to regulate Pol I and Pol II transcription. From a broader perspective, the similarities in nucleoplasmic and nucleolar transcriptional regulatory may be indicative of the proposed co-evolution of the nucleus and nucleolus (Lo *et al.* 2006). It has been demonstrated that single rDNA repeats are capable of forming mini-nucleoli (Karpen *et al.* 1988; Scheer and Hock 1999); however, only tandemly repeated rDNA arrays form proper nucleoli (Scheer and Hock 1999). Thus, it may be postulated that early in the evolution of the nucleus, the few copies of rDNA in the genome were not contained within any subnuclear structure; however, as genomes became more complex and cellular demand for protein synthesis increased, it became favorable to densely cluster a large number of rDNA repeats. This may be reflected in the current understanding of the nucleolus as “an organelle formed by the act of building a ribosome” (Mélèse and Xue 1995). Thus, the nuclear regulatory machinery that had participated in modulating rRNA transcription before the emergence of the nucleolus continued to function in this capacity in this novel subnuclear compartment. Although this hypothesis is speculative, further studies into the evolution of transcriptional regulation and the nucleolus will undoubtedly reveal striking insights into these dual-function factors.

## Supplementary Material

Supporting Information
