# Supplementary material for: Genomic Characterization of the Mouse Ribosomal DNA Locus
Source: G3 (Bethesda). 2013 Dec 17;4(2):243–54. doi: 10.1534/g3.113.009290 (PMC3931559; doi:10.1534/g3.113.009290)
Supplement: Supporting Information [file supp_4_2_243__index.html]

Genomic Characterization of the Mouse Ribosomal DNA Locus — Supporting Information 

# Genomic Characterization of the Mouse Ribosomal DNA Locus

## Supporting Information for Zentner, Balow, and Scacheri, 2014

**Files in this Data Supplement:**

- Supporting Information - Figures S1-S2 and Tables S1-S3 (PDF, 471 KB)
- Figure S1 - ChIP-PCR analysis of OCT4 binding to rDNA in mESCs. (PDF, 263 KB)
- Figure S2 - Negative controls for chromain-binding protein association with rDNA in mESCs. (PDF, 244 KB)
- Table S2 - Primers used for ChIP-PCR assays. (PDF, 187 KB)
- Table S3 - Primers used for qRT-PCR assays. (PDF, 297 KB)
- Table S1 - Mappability values of mouse rDNA as determined by BEADS (.xls, 5 MB)
