# Supplementary material for: Genomic Characterization of the Mouse Ribosomal DNA Locus
Source: G3 (Bethesda). 2013 Dec 17;4(2):243–54. doi: 10.1534/g3.113.009290 (PMC3931559; doi:10.1534/g3.113.009290)
Supplement: Supporting Information [file supp_g3.113.009290_009290SI.pdf]

G3 · Genes | Genomes | Genetics

# Genomic characterization of the mouse ribosomal DNA locus

Gabriel E. Zentner[*,1], Stephanie A. Balow*, and Peter C. Scacheri[*,§]

[*]Department of Genetics and Genome Sciences, Case Western Reserve University, 2109 Adelbert Rd., Cleveland, OH 44106, USA
[§]Comprehensive Cancer Center, Case Western Reserve University, 11000 Euclid Ave., Cleveland, OH 44106, USA
[1]Current address: Basic Sciences Division, Fred Hutchinson Cancer Research Center, 1100 Fairview Ave. N., Seattle, WA 98109, USA

Correspondence to Peter C. Scacheri
BRB 627
Case Western Reserve University
2109 Adelbert Rd.
Cleveland, OH 44106, USA
Email: pxs183@case.edu
Tel: 216-368-3458
Fax: 216-368-3432

**DOI: 10.1534/g3.113.009290**

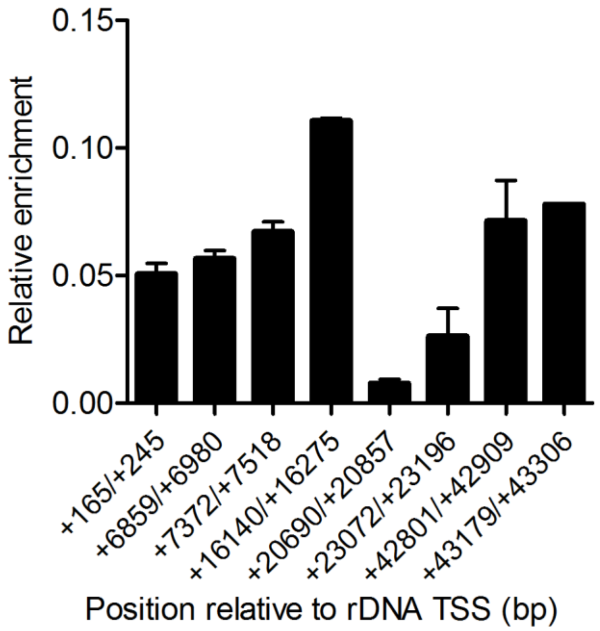


**Figure S1** ChIP-PCR analysis of OCT4 binding to rDNA in mESCs.

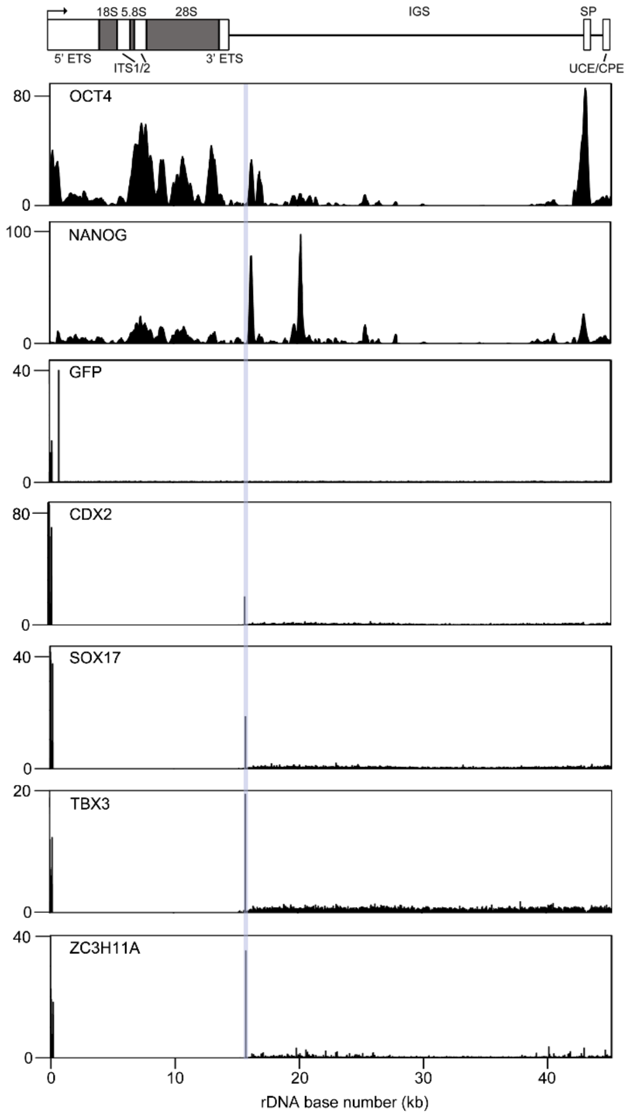


**Figure S2** Negative controls for chromain-binding protein association with rDNA in mESCs. Nuclear-targeted GFP as well as four transcription factors fail to bind rDNA in mESCs, indicating that the association of pluripotency factors with rDNA is specific. OCT4 and NANOG rDNA ChIP-seq profiles are shown for comparison.

**Table S1 Mappability values of mouse rDNA as determined by BEADS.**

Available for download as an Excel file at http://www.g3journal.org/lookup/suppl/doi:10.1534/g3.113.009290/-/DC1

**Table S2   Primers used for ChIP-PCR assays**

| Amplicon relative to rDNA TSS (bp) | Forward primer (5' - 3') | Reverse primer (5' - 3') |
|---|---|---|
| +165/245 | CTTGCGTGTGCTTGCTGT | GAAATCGGGAAAAACGTCTG |
| +6859/+6986 | ACACCCGAAATACCGATACG | AGTGCGTTCGAAGTGTCGAT |
| +7372/+7518 | CATCTGCTCTGGTCGAGGTT | GCAAGACCCAAACACACACA |
| +16140/+16275 | CCCTTTTACACCTCCCCCTA | TTTCCAAAGGGCTACATTGC |
| +20690/+20857 | GGAATCCGATGCACACTTTT | TGTGTGTGTGTGTGTGTGTGA |
| +23072/+23196 | AATACGCCGTGTGTGTGTGT | CAATGTGCCTTCATGATCAAA |
| +42801/+42909 | AACTGTGCCTGTTCCCTCAC | GGCACCCAAAAACGAAAGTA |
| +43179/+43306 | GACACAGGAGAGGGAAGTGC | CTCCCTGTACGACCTCCTTG |

**Table S3 Primers used for qRT-PCR assays**

| Target | Forward primer (5' - 3') | Reverse primer (5' - 3') |
|---|---|---|
| pre-rRNA | TGTCTGCCCGTATCAGTAACTGTC | CCCTGGCCCGAAGAGAACT |
| *GAPDH* | AAGGTCATCCCAGAGCTGAA | AGACAACCTGGTCCTCAGTGTAG |
