# Supplementary figures and images for: Genomic Characterization of the Mouse Ribosomal DNA Locus
Source: G3 (Bethesda). 2013 Dec 17;4(2):243–54. doi: 10.1534/g3.113.009290 (PMC3931559; doi:10.1534/g3.113.009290)

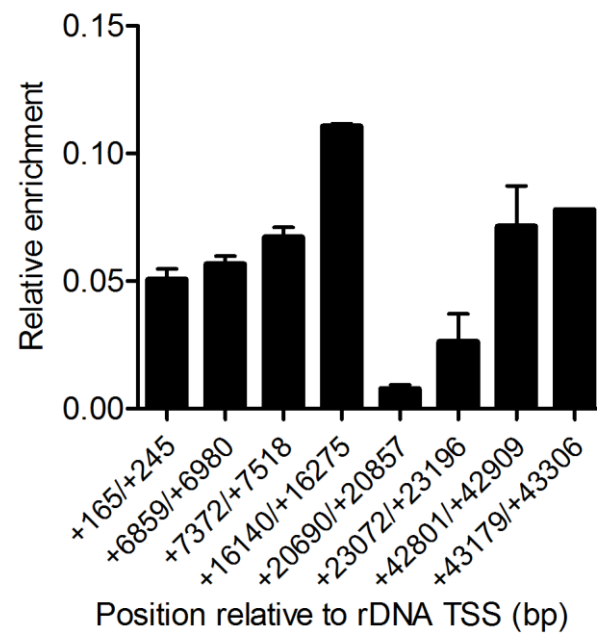

**Figure S1** ChIP-PCR analysis of OCT4 binding to rDNA in mESCs.

Supplement: Supporting Information [file supp_g3.113.009290_FigureS1.pdf]
