# Supplementary material for: Genomic Characterization of the Mouse Ribosomal DNA Locus
Source: G3 (Bethesda). 2013 Dec 17;4(2):243–54. doi: 10.1534/g3.113.009290 (PMC3931559; doi:10.1534/g3.113.009290)
Supplement: Supporting Information [file supp_g3.113.009290_FigureS2.pdf]

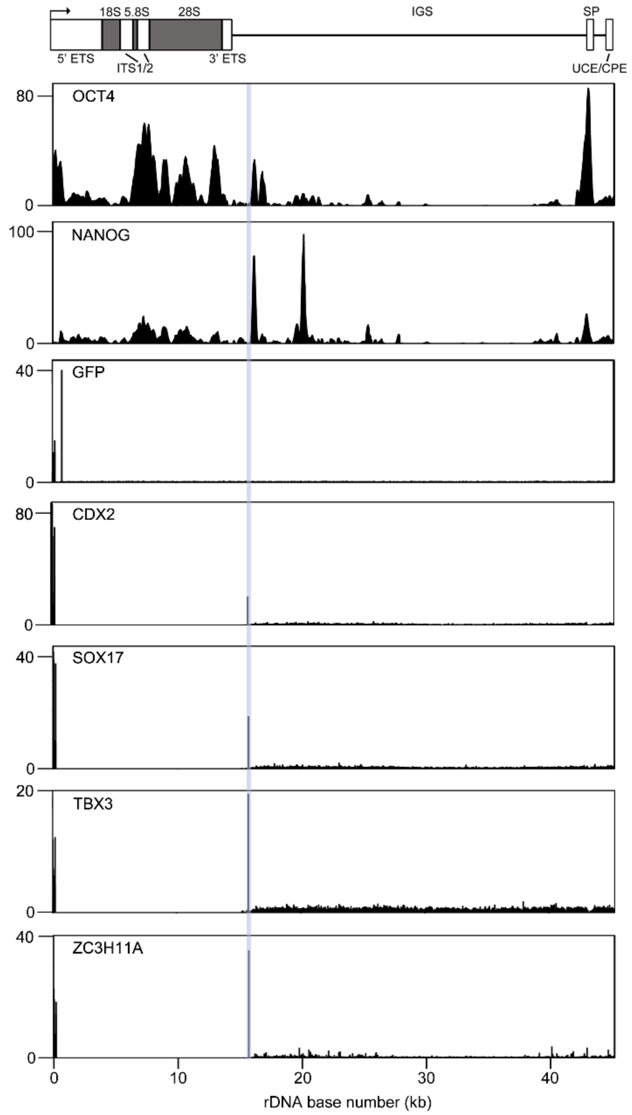

Figure S2 Negative controls for chromain-binding protein association with rDNA in mESCs. Nuclear-targeted GFP as well as four transcription factors fail to bind rDNA in mESCs, indicating that the association of pluripotency factors with rDNA is specific. OCT4 and NANOG rDNA ChIP-seq profiles are shown for comparison.
