# Supplementary material for: Genomic Characterization of the Mouse Ribosomal DNA Locus
Source: G3 (Bethesda). 2013 Dec 17;4(2):243–54. doi: 10.1534/g3.113.009290 (PMC3931559; doi:10.1534/g3.113.009290)
Supplement: Supporting Information [file supp_g3.113.009290_TableS2.pdf]

Table S2 Primers used for ChIP-PCR assays

Amplicon relative to rDNA TSS (bp)	Forward primer (5' - 3')	Reverse primer (5' - 3')
+165/245	CTTGCGTGTGCTTGCTGT	GAAATCGGGAAAAACGTCTG
+6859/+6986	ACACCCGAAATACCGATACG	AGTGCGTTCGAAGTGTCGAT
+7372/+7518	CATCTGCTCTGGTCGAGGTT	GCAAGACCCAAACACACACA
+16140/+16275	CCCTTTTACACCTCCCCCTA	TTTCCAAAGGGCTACATTGC
+20690/+20857	GGAATCCGATGCACACTTTT	TGTGTGTGTGTGTGTGTGTGA
+23072/+23196	AATACGCCGTGTGTGTGTGT	CAATGTGCCTTCATGATCAAA
+42801/+42909	AACTGTGCCTGTTCCCTCAC	GGCACCCAAAAACGAAAGTA
+43179/+43306	GACACAGGAGAGGGAAGTGC	CTCCCTGTACGACCTCCTTG
