# Supplementary material for: Genomic Characterization of the Mouse Ribosomal DNA Locus
Source: G3 (Bethesda). 2013 Dec 17;4(2):243–54. doi: 10.1534/g3.113.009290 (PMC3931559; doi:10.1534/g3.113.009290)
Supplement: Supporting Information [file supp_g3.113.009290_TableS3.pdf]

Table S3   Primers used for qRT-PCR assays

Target	Forward primer (5' - 3')	Reverse primer (5' - 3')
pre-rRNA	TGTCTGCCCGTATCAGTAACTGTC	CCCTGGCCCGAAGAGAACT
GAPDH	AAGGTCATCCCAGAGCTGAA	AGACAACCTGGTCCTCAGTGTAG
